# Factors influencing changes in health related quality of life of caregivers of persons with multiple chronic conditions

**DOI:** 10.1186/s12955-016-0486-7

**Published:** 2016-05-27

**Authors:** Wendy Duggleby, Allison Williams, Sunita Ghosh, Heather Moquin, Jenny Ploeg, Maureen Markle-Reid, Shelley Peacock

**Affiliations:** Faculty of Nursing, University of Alberta, 3rd Level ECHA, 11405 - 87th Ave, Edmonton, AB T6G 1C9 Canada; School of Geography and Earth Sciences, McMaster University, 1280 Main Street West, Hamilton, ON L8S 4K1 Canada; Alberta Health Services-Cancer Care, University of Alberta, Edmonton, AB Canada; Faculty of Nursing, University of Alberta, 3rd Level ECHA, 11405 - 87th Ave, Edmonton, AB T6G 1C9 Canada; School of Nursing, McMaster University, Room HSc3N25C, 1280 Main Street West, Hamilton, ON L8S 4K1 Canada; School of Nursing, McMaster University, 1280 Main Street West, Hamilton, ON L8S 4K1 Canada; College of Nursing, University of Saskatchewan, E-Wing, 4340, 104 Clinic Place, Saskatoon, SK S7N 2Z4 Canada

**Keywords:** Caregivers, Quality of life, General self efficacy, Gender identity

## Abstract

**Background:**

The majority of care for older adults with multiple chronic conditions (MCC) is provided by family (including friends) caregivers. Although caregivers have reported positive benefits to caregiving they also experience decreases in their physical and mental health. As there is a critical need for supportive interventions for this population, it is important to know what influences the health of family caregivers of persons with MCC. This research examined relationships among the changes from baseline to 6 months in health related quality of life (SF12v2) of family caregivers caring for older adults with multiple chronic conditions and the following factors: a) demographic variables, b) gender identity [Bem Sex Role Inventory (BSRI)] c) changes in general self-efficacy [General Self Efficacy Scale (GSES) (baseline to 6 months) and d)) changes in caregiver burden [Zarit Burden Inventory (ZBI)] baseline to 6 months. Specific hypothesis were based on a conceptual framework generated from a literature review.

**Methods:**

This is a secondary analysis of a study of 194 family caregivers who were recruited from two Canadian provinces Alberta and Ontario. Data were collected in-person, by telephone, by Skype or by mail at two time periods spaced 6 months apart. The sample size for this secondary analysis was *n* = 185, as 9 participants had dropped out of the study at 6 months. Changes in the scores between the two time periods were calculated for SF12v2 physical component score (PCS) and mental component score (MCS) and the other main variables. Generalized Linear Modeling was then used to determine factors associated with changes in HRQL.

**Results:**

Participants who had significantly positive increases in their MCS (baseline to 6 months) reported lower burden (ZBI, *p* < 0.001), and higher general self-efficacy (GSES, *p* < 0.001) and Masculine BSRI (*p* = 0.025). There were no significant associations among variables and changes in PCS (baseline to 6 months).

**Conclusions:**

Our findings suggest that a masculine gender identity (which incorporates assertive and instrumental approaches to caregiving), and confidence in the ability to deal with difficult situations was positively related to improvement in mental health for caregivers of persons with MCC. Decreases in perceptions of burden in this populations was also associated with improvements in mental health. Further research is needed to explore ways to support caregivers of older persons with multiple chronic conditions living at home.

## Background

With increasing numbers of persons over the age of 65 there is an increasing demand in Canada for family/friends to provide care in the home [1]. This care is complex as the prevalence of multiple chronic conditions (at least two chronic physical or behavioral health problems that have lasted 6 months or longer) increases with age [2]. The care provided by family/friends is critical for maintaining the health of Canadians older adults with chronic health conditions [1]. However, caring for older adults impacts the daily lives of caregivers and can result in adverse physical and psychological health outcomes. For example, caregivers experiencing caregiver burden and strain have a 63 % higher risk of mortality than non-caregivers [3], which in many cases is greater than the risk for care recipients [4]. There are large gaps in knowledge regarding family caregivers of an increasing population of older adults with MCC [5]. Identifying factors impacting the lives of caregivers for older adults with MCC will help to inform appropriate supportive interventions for this vulnerable population.

Very little research has focused specifically on the health related quality of life (HRQOL) of family caregivers providing care to individuals with MCC. However, in general, caring for patients with multiple comorbidities has been associated with increased burden and poor HRQOL for caregivers [4, 6, 7]. Among caregivers of persons with long-term neurological conditions, HRQOL is poorer when the person they are caring for has more severe symptoms, or depression, or if the amount of time providing care increases [8]. Heuvel et al. suggested that caregiver stress is increased by the severity of chronic condition [9]. Along with the specific challenges encountered in providing care for those with particular chronic conditions, caregivers of persons with MCC face corresponding complexities that they must negotiate with their care partners. These include *siloed* health care [10] as well as potential disease progression at faster rates, difficulties in diagnosing new conditions and interactions between treatments for different conditions [5].

### Caregiver demographics and health related quality of life

Many caregivers’ characteristics also are associated with their HRQOL. For example women who are caregivers reported lower well-being or health status, particularly as related to mental health, [11–14] and experienced higher levels of depression than men [7, 14–18]. Male caregivers often reported higher levels of well-being, both physically [16] and mentally [16, 19] than female caregivers. Moreover, female caregivers also tend to be at a greater risk for comorbidities and chronic illnesses, regardless of the care recipient’s disease [20, 21].

The age of the caregiver, may also be associated with their HRQOL. Older caregivers of persons with stroke or dementia were found to more likely be living with chronic illnesses compared to their younger counterparts [22–26]. The physical health of older caregivers can be compromised by caregiving demands, biological vulnerabilities associated with age, and psychological factors such as feelings of loss and distress [7]. Contributors to poor HRQOL among caregivers of patients with a variety of chronic diseases also include: having a lower monthly income [4] and being a spouse of the care recipient [4, 27].

### Gender identity and caregiver health related quality of life

Studying gender roles in relation to caregiving and HRQL is important as both women and men spouses have been found to cross gender role boundaries when providing care for their partners by doing tasks originally undertaken by their spouses [28–30]. Caregiving has been viewed predominantly as a feminine role within society [28]. Because of the focus on caring as ‘women’s’ work, a number of studies have considered how men negotiate this stereotypically female role and the ‘feminine’ traits associated with caregiving [28]. However, in a study of gender identity of widowed male those who scored higher on the masculine subscale of the Bem Sex Roles Inventory (BSRI) reported higher psychological well-being [29]. This suggested that masculine approaches to caregiving were associated with higher mental health HRQOL. However no reported studies have examined gender identity and its relationship to the HRQOL of caregivers of persons with MCC.

### Self-efficacy and caregiver health related quality of life

General self-efficacy is defined as confidence in the ability to deal with difficult situations [31]. Literature related to self-efficacy concerning HRQL of caregivers of patients with multiple comorbidities is lacking. However, self-efficacy has been found to be related to higher quality of life in caregivers of persons with cancer [32]. Self-efficacy is inversely associated with depression in caregivers of persons with dementia [33–38]. Although the association of self-efficacy with HRQOL has not been reported in caregivers of persons with MCC, it may be associated with their mental HRQOL.

### Caregiver burden and health related quality of life

Caregiver burden can be described as the hardships associated with caring for a person who is often suffering from a medical condition [39]. Depression among caregivers has also been closely linked with their perceived burden [6, 40–44]. Moreover caregiver burden has been found consistently to be inversely associated with caregiver quality of life [25, 45–47].

Much of the research on family caregiving has focused on caregiver burden [48, 49]. Burden, however, is not as inclusive a measure [49, 50] and may offer an overly negative picture of caregiver experience [49, 51]. As well it does not reflect the health status of the caregivers, although burden has been found to be associated with quality of life [49, 51–54]. McConaghy and Caltabiano [55] found that predictors for psychological and physical well-being among dementia caregivers included perception of higher burden. This finding suggested a negative association between burden and HRQOL [55]. It is unknown if this relationship between burden and HRQOL changes over time. The studies of caregivers’ HRQL have typically been cross-sectional and not examined factors influencing changes in HRQOL of caregivers of persons with MCC.

### Purpose

Overall the literature suggests that demographic characteristics, gender identity, general self-efficacy and burden may influence the HRQOL of caregivers of older persons with MCC. Gaining greater understanding of factors impacting HRQOL of family caregivers has been recognized as a priority for comprehensive health care for both patients who are ill and their family caregivers [3]. This study reports the results of a secondary analysis of data from a study whose primary purpose was to investigate how social location (e.g., sex, gender, age, education) of family caregivers of older adults with MCC impact caregiver burden overtime [56]. The purpose of the secondary analysis reported herein, was to examine relationships among the changes from baseline to 6 months in health related quality of life (SF12v2) of family caregivers caring for older adults with multiple chronic conditions and the following factors: a) demographic variables, b) gender identity [Bem Sex Role Inventory (BSRI)] c) changes in general self-efficacy [General Self Efficacy Scale (GSES) (baseline to 6 months) and d)) changes in caregiver burden [Zarit Burden Inventory (ZBI)] baseline to 6 months. Based on the review of the literature the following were the hypotheses for the secondary analysis:

1. Demographic variables (caregiver sex, age, relationships) and caregiver gender identity scores would have a significant association with changes in caregivers’ HRQOL scores from baseline to 6 months,
2. Changes in caregiver general self-efficacy scores from baseline to 6 months would have a positive significant association with participants’ change in HRQOL scores from baseline to 6 months.
3. Changes in caregiver burden scores from baseline to 6 months would be significantly and negatively associated with changes in caregivers’ HRQOL scores from baseline to 6 months.

## Methods

This study is a quantitative secondary analysis of data from a study of caregivers of persons with MCC in Alberta and Ontario Canada. The original study used a mixed methods repeated - measures design. Details of this study are reported elsewhere [56]. The original study received ethical approval from the University of Alberta and McMaster University Research Ethics Review Boards.

Using convenience sampling 194 participants were recruited to the original study through a variety of means. Inclusion criteria for study participants were as follows: a) informal caregivers actively providing care to an older adult (65 or older) with MCC living in the community, b) 18 years of age or older, and c) English speaking. Informal caregivers were defined as family and or friends providing any form of assistance to an older persons (65 years of age and older) with MCC (two or more) living in the community. Exclusion criteria were as follows: a) paid caregivers, b) caregiving for a person who was residing in an institution, and c) non-English speaking.

Participants completed questionnaires between July 2013 and June 2014 at two time periods 6 months apart. A subset of 40 participants completed qualitative interviews. Following obtaining consent, demographic information was collected. Twice (6 months apart) caregivers were asked to complete the SF-12v2, the General Self Efficacy Scale, the BEM Sex Role Inventory and the Zarit Burden Inventory. A description of the measures are below:

### 12-item short-form health survey (SF-12v2) (dependent variable)

The SF12v2 is a shortened version of the 36-item Short Form Health Survey (SF-36) and has been used previously to measure caregiver health related quality of life (HRQOL) [57]. The SF-12v2 consists of 12 questions, with two to six response level options, depending on the question [58]. Both a Physical Component Summary (PCS) and Mental Component Summary (MCS) are produced from the SF-12, with a higher score indicating better physical or mental health, and therefore an overall improved perceived HRQOL. The MCS and PCS have been shown to have high internal consistency (α = 0.80) [59]. Test-retest reliability for the PCS was (*r* = 0.78) and for the MCS *r* = 0.60) [59]. The SF-12v2 PCS and MCS convergent validity with the SF 36 was *r* = 0.95 and 0.96 [60].

### General self-efficacy scale (GSES)

The GSES is a measure of a persons’ confidence in their ability to deal with novel, adverse and difficult situations [61]. The GSES is a Likert-type scale with 10 items scored using a 4-point scale (1 “not true at all” to 4 “exactly true”) which are summed to produce a total score (minimum 10 and maximum 40). A higher score indicates that a participant is feeling greater self-efficacy. The GSES has been found to be a reliable and valid measure across a number of populations and countries [59] with internal consistency ranging from α = 0.75– 0.91. Test-retest reliability has been found to be *r* = 0.82 [62].

### Zarit burden interview (ZBI)

The ZBI is a 12-item reliable (r = 0.92) and valid measure of caregiver burden [63]. The items are scored using a 5-point Likert scale (1 “never” to 5 “nearly always”) with higher scores indicating greater burden [64].

### Bem Sex role inventory (BSRI)

The BSRI is a commonly used measure of gender role that has internal consistency (α = 0.80–,82) and high test retest reliability (*r* = 0.89) and has been validated with populations from many cultures and age groups [65]. It consists of 30 personality characteristics on which respondents are asked to rate themselves on a 7-point Likert scale ranging from 1 (never or almost never true) to 7 (almost or almost always true) [66]. Ten items on the scale reflect masculine personality traits such as assertive, strong personality and dominant. For the feminine personality traits, 10 of the items reflect traits such affectionate, sympathetic and sensitive to others. Examples of the 10 neutral items reflect conscientious, and unpredictable personality traits [66]. The scores are presented as mean and standard deviations in each category for the participants. The maximum score in each category is 70.

### Secondary data analysis

A power analysis for the secondary data analysis was calculated post hoc, using Cohen’s [67] formula for calculating sample size. A medium effect size (0.15) and a power of 80 % for regression with six independent variables (age, sex, relationships, gender identity, general self-efficacy, and caregiver burden), the required sample size was 96. The dependent variables were participants’ mental (SF12v2MCS) and physical (SF12v2PCS) HRQOL.

All data had been entered into SPSS v22 and anonymized. Using descriptive statistics mean and standard deviation scores for the main variables were calculated. Frequency and proportions were reported for categorical variables. No issues were noted regarding multi-collinearity.

To address the three hypotheses, changes (baseline to 6 months) for continuous variables (GSES, ZBI, SF12 MCS, and PCS) were calculated. General linear modeling (GLM) was utilized to determine what factors predicted changes in HRQL (baseline and 6 months). GLM is an extension of linear regression method and can handle categorical, count and continuous data as the response variable [68]. Each independent variable (age, sex, and relationships, BSRI, and changes in GSES and ZBI) was entered separately for the univariate analysis. Those variables that were significant at the *p* ≤ 0.10 were included into the multivariate model as well as the hypothesized variables: age, sex, relationships, BSRI, and change in GSES and ZBI. A *p*-value ≤ 0.05 was used for statistical significance, unless otherwise stated and two sided tests were used for all comparisons.

## Results

### Participants

One hundred and ninety-four participants were enrolled in the study and completed the first data collection. Nine participants (4.6 %) did not participate in the second data collection for a variety of reasons (e.g. overwhelmed with caregiving, person they were caring for was admitted to long term care or died, unable to be reached). Only those who completed baseline and 6 months data were included in this secondary analysis (*n* = 185).

The majority of participants (83.2 %) were female (*n* = 154) and over the age of 65 (*n* = 116; 60 %). Eighty participants (43 %) were spouses and 105 (57 %) had other relationships with the care recipient (e.g. sons, daughters, and friends). Participants were caring for people who had a mean of 6.17 (SD = 2.78, range 2–14) chronic conditions. They had been caregiving on average 77.17 months (SD = 87.9, range 3–613). Participants reported a higher score in the BSRI feminine category (mean 59.93, SD = 6.64) than other BSRI categories (Table 1). Table 1 describes other demographic characteristics.

**Table 1:** Caregiver Demographic Characteristics and BSRI Scores *n* = 185

Variables	Categories	Frequency	Percentage
Age (years)	65+	127	68.6
	>65	58	31.4
Sex	Female	154	83.2
	Male	31	16.8
Ethnicity/Race	Caucasian	167	90.3
	Chinese	6	3.2
	Black	2	1.1
	Filipino	2	1.1
	Arab	1	0.5
	South East Asia	1	0.5
	Other	6	3.2
Marital Status	Single	28	15.1
	Married	127	68.6
	Widowed	4	2.2
	Divorced/separated	18	9.7
	Other	8	4.3
Employed	Yes	66	35.7
	No	117	63.2
Annual Income ($)	>10,000	2	1.1
	10,000–19,999	9	4.9
	20,000–29,999	16	8.6
	30,000–39,999	21	11.4
	40,000–49,999	25	13.5
	50,000–59,999	13	7.0
	60,000–69,999	10	5.4
	70,000 or more	58	31.4
	Prefer not to answer	31	16.8
Finances meet needs	Yes	119	63.2
	No	66	35.7
Education	Post-secondary education	133	71.8
	Apprenticeships	4	2.0
	No secondary education	48	25.9
Residence	Urban	174	94.1
	Rural	11	5.9
Relation to recipient	Husband/wife/partner	80	43.1
	Parent	7	3.3
	Son/daughter	83	44.9
	Sister/brother	2	1.1
	Other	13	7.0
Variable		X (SD)	Range
BSRI (Time 1)	Masculine	49.05 (9.07)	27–67
	Feminine	59.93 (6.64)	41–70
	Neutral	45.57 (4.30)	32–57

The mean, standard deviations and range of the ZBI, GSES, SF12 PCS and MCS at baseline and six months and change scores are presented in Table 2. When comparing the PCS scores at baseline and 6 months to Canadian SF36 normative values [69], both scores were below the Canadian average of 50.5 (SD = 9.0). In regards to the MCS at baseline and 6 months both scores were also below the Canadian average of 51.7 (SD = 9.1).

**Table 2:** Means, Standard Deviations atBaseline and 6 months and Changes (Baseline to Six Months)

Measure	N	Baseline Mean (SD) (Range)	6 monthsMean (SD) (Range)	Change Mean (SD) (Range)
Zarit Burden Inventory-Total Score	185	21.27 (9.14) (1.00–47.00)	19.63 (9.16) (0.00–43.00)	1.60 (6.24) (–21.00–17.00)
General Self-Efficacy Scale (GSES) - Total Score	185	32.31 (4.33) (18.00–40.00)	32.33 (4.54) (16.00–40.00)	–0.15 (4.06) (–10.00–12.00)
SF12 Physical Component (PCS)	185	48.83 (9.49) (23.57–66.02).	48.68 (9.78) (21.03–66.46)	−0.02 (7.44) (−26.44–20.64)
SF12 Mental Component (MCS)	185	44.12 (10.42) (21.55–70.21))	43.33 (11.52) (11.21–66.75)	0.63 (9.89) (−28.99–28.59)

### Factors influencing changes in physical component score (SF12v2) baseline to Six months

Using univariate analysis none of the variables had a significant association with change in PCS, as the dependent variable. While not significant at *p* < 0.10 in the univariate analysis, the following variables were included in the final model based on the literature: caregiver age, sex, and relationship to the care recipient and changes in the caregiver rated scores for GSES, ZBI, and BSRI. The results of the multivariate analysis (Table 3) suggested that there were no significant factors influencing changes in PCS scores (baseline to 6 months).

**Table 3:** Multivariate Analysis Change in SF12V2 PCS from Baseline to Six Months

Parameter	B	Std. Error	95 % Wald Confidence Interval		Hypothesis Test		
			Lower	Upper	Wald Chi-Square	df	Sig.
(Intercept)	−5.004	7.046	−18.814	8.806	0.504	1	0.478
Caregiver_ Sex Male	−0.023	1.543	−3.047	3.002	0.000	1	0.988
Caregiver Sex = Female	0^a^						
Caregiver age <65	0.891	1.617	−2.278	4.059	0.303	1	0.582
Caregiver age ≥65	0^a^						
Relationship spouse/partner	−0.187	1.565	−3.253	2.879	0.014	1	0.905
Relationship [other]	0^a^						
Change General Self Efficacy Scale (baseline – six months)	0.117	0.139	-.0156	0.390	0.710	1	0.399
Change Zarit Burden Inventory (baseline-six months)	0.105	0.087	−0.067	0.276	1.426	1	0.232
Masculine Bem Sex Role Inventory	−0.027	0.063	−0.150	0.097	0.177	1	0.674
Feminine Bem Sex Role Inventory	−0.003	0.087	−0.174	0.169	0.001	1	0.974
Neutral BSRI	0.127	0.135	−0.137	0.392	0.893	1	0.345
(Scale)	54.078	5.653	44.059	66.375			

^a^reference category

### Factors influencing changes mental component scores (SF12v2) baseline to Six months

In the univariate analysis, with change in MCS scores (baseline to 6 months) as the dependent variable, the following variables were statistically significant: Changes in GSES (*p* < 0.001) and ZBI (*p* < 0.001) scores (baseline to 6 months) and Masculine BSRI (*p* = 0.025). No other variables were significant. The variables significant at the univariate analysis stage, as well as the hypothesized variables of age, sex, relationship and masculine, feminine and neutral BSRI, were entered into the multivariate model (presented in Table 4). Participants who had significantly positive increases in their MCS reported lower burden (ZBI, *p* < 0.001), and higher general self-efficacy (GSES, *p* < 0.001) and Masculine BSRI (*p* = 0.025) changes in scores.

**Table 4:** Mulitvariate Analysis with Change in SF12v2 MCS (Baseline to Six Months) as the Dependent Variable

Parameter	B	Std. Error	95 % Wald Confidence Interval		Hypothesis Test		
			Lower	Upper	Wald Chi-Square	df	Sig.
(Intercept)	−11.722	8.034	−27.468	4.025	2.129	1	0.145
Caregiver Sex = male	−2.413	1.759	−5.861	1.036	1.880	1	0.170
Caregiver Sex = female	0^a^						
Caregiver age < 65	−2.317	1.843	−5.930	1.296	1.580	1	0.209
Caregiver age ≥ 65	0^a^						
Relationship spouse/partner	−0.208	1.7839	−3.704	3.288	0.014	1	0.907
Relationship [other]	0^a^						
Change General Self-efficacy (baseline to six months)	0.728	0.159	0.417	1.040	21.019	1	0.001^*^
Change Zarit Burden Inventory (baseline to six months)	−0.570	0.099	−0.765	−0.374	32.556	1	0.001^*^
Masculine Bem Sex Role Inventory	0.162	0.072	0.020	0.303	5.027	1	0.025^*^
Feminine Bem Sex Role Inventory	0.079	0.099	−0.117	0.274	0.621	1	0.431
Neutral Bem Sex role Inventory	0.058	0.154	−0.243	0.359	0.142	1	0.706
(Scale)	70.309	7.3502	57.283	86.297			

^a^reference

^*^Significant ≤0.05

## Discussion

The purpose of this study was to identify factors that significantly influenced the changes in health related quality of life of caregivers caring for older persons with MCC. Changes from baseline to 6 months in mental health were significantly associated with burden, general self-efficacy and masculine gender identity. None of the variables were significantly associated with changes in physical health.

Increases in mental health were associated with decreases in burden. This is similar to the results from other studies as caregiver burden has been consistently found to be inversely associated with caregiver mental health [6, 40–44]. However, changes in burden scores were not a significant predictor of changes in physical health as we had hypothesized, suggesting its influence may be limited to the mental health of caregivers of persons with MCC. Another possible explanation is that the majority of our participants (56.7 %) were not spouses. In a meta-analysis study of factors influencing the physical health of caregivers, Pinquart and Sorenseon [70] suggested that burden was more strongly associated with spousal caregivers’ physical health than in non-spousal caregivers. As well our time frame of 6 months might be too short to identify significant changes and additional patient factors such as functional status were not controlled. This is the first study that we know of to look at factors that influence changes in health related quality of life in caregivers of older persons with MCC living in the community. More research is needed to examine why changes in burden scores baseline to 6 months were not significantly associated with physical health.

Masculine gender identity had a significant influence on improvements in mental health. which is similar to the findings of a study of family caregivers that found masculine gender identity was significantly associated with psychological well-being [29]. Our findings suggested that a masculine gender identity, which includes personality traits of assertiveness, independence, and self-sufficiency, was positively associated with improvements in participants’ mental health. However, the literature suggests that when undertaking the role of caregiver, men renegotiate their conceptions of masculinity tempering the more dominant masculine personality trait with feminine traits of empathy and compassion [66]. It is unclear from the literature, however, when this renegotiation may occur and our findings suggest that assuming the personality traits associated with a feminine role identity may have unintended consequences of decreasing caregivers’ mental health. Our participants reported more feminine traits than masculine, which maybe one explanation for their physical and mental health being below the average Canadian. None of the categories of gender role identify were found to be significantly associated with changes (declines or improvements) in physical health. More research is needed to determine the association of gender identity and physical health and why masculine gender identity is significantly associated with improvements in caregivers’ mental health. There is at this time a paucity of research with respect to gender identity and caregivers’ of persons with MCC health related quality of life. Future research should consider further exploration into this area.

Increases (baseline to 6 months) in general self- efficacy were a significant predictor for improvements in mental health. The finding of the significant positive relationship of general self -efficacy (the confidence in their ability to deal with difficult situations) with mental health is similar to the findings from other studies. In particular caregivers of persons with dementia have reported a significant negative relationship between general self-efficacy and depression [33–38]. Self -efficacy has been found to help control upsetting thoughts experienced by burdened and distressed caregivers [71, 72]. We had also hypothesized positive changes in general self-efficacy scores would predict improvements in physical health. In contrast, in a review of cognitive behavioral interventions, Graves found that interventions, focusing on a component of Social Cognitive Theory (increasing self-efficacy) had an influence on a person’s psychological and physiological functioning (health status) [73]. It is unclear why positive changes in general self-efficacy scores did not also increase physical health. Further research is needed to determine the mechanisms through which positive changes in self-efficacy influences improvements in mental health and not in physical health.

When compared to Canadian normative health related quality of life [69], the participants’ physical and mental health at baseline and 6 months were lower than the norms. These findings underscore the concerns regarding caregivers’ physical and mental health as their poor HRQOL was evident at baseline and 6 months later. More research is needed to examine trends over time in the HRQOL of caregivers of persons with MCC as most of the research conducted with caregivers has been cross -sectional.

### Limitations

There are several limitations associated with this study in regards to sample and methodology. The majority of the sample was Caucasian although some participants represented different ethnicities. There are limitations for the generalizability of the findings. For example as multiple methods of recruitment were used the response rate could not be calculated and a convenience sampling approach was used. Another limitation was data were only collected at two points in time. There may be more fluctuation in the scores that would be captured if data were collected more frequently. Moreover, very little information was collected about the care recipient which may also have a significant impact on caregiver quality of life.

Another limitation of the study that may have influenced the findings was the use of a health related quality of life tool (SF12v2). Although the SF12v2 is a well-established measure it has been criticized for not measuring important aspects of quality of life for caregivers such as relationships and existential quality of life [74]. Thus the use of another HRQOL measure may have resulted in different findings. In review of the literature of instruments used to measure caregivers’ HRQOL the authors concluded that diverse instruments have been used with diverse outcomes [75].

## Conclusions

The study findings suggest that caregivers of older persons with multiple chronic conditions who have positive changes in their general self-efficacy, decreases in their perception of burden and identify with a masculine gender identity role, have positive changes in their mental health. This suggests that health care providers should consider ways to support caregivers increase their confidence in their ability to deal with difficult situations and decrease perceptions of caregiver burden.

Future research should examine ways to decrease perceptions of burden and increase caregiver’s confidence in their ability to problem solve difficult situations. Understanding the best strategies to accomplish this would be important information needed to support caregivers of older persons with MCC. More research is also needed to examine what influences changes in health related quality of life over time. By looking at the changes, researchers and health care professionals can understand the best way to bring about improvements over time in physical and mental health for this vulnerable group of caregivers. As such the findings of this study are a foundation for future research.
